# Effects of brain contusion on mild traumatic brain injured patients

**Published:** 2012-11

**Authors:** Mohammad Amin Zare, Koorosh Ahmadi, Shayan Abdollah Zadegan, Davood Farsi, Vafa Rahimi-Movaghar

**Affiliations:** ^a^Department of Emergency Medicine, Tehran University of Medical Sciences, Tehran, Iran.; ^*b*^Department of Emergency Medicine, Alborz University of Medical Sciences, Alborz, Iran.; ^*c*^Sina Trauma and Surgery Research Center, Tehran University of Medical Sciences, Tehran,Iran.

## Abstract

**Background:**

Traumatic brain injury (TBI) is an important health issue with high prevalence worldwide. The most common type of TBI is mild traumatic brain injury (MTBI). MTBI is defined as a condition with self-limited symptoms; however it could cause some structural abnormalities in brain and become more complicated. Visible structural brain damage could have an important effect on post- MTBI recovery, but the outcome is not yet fully understood. Therefore, the present study aims to investigate the clinical course of MTBI patients whose computed tomography (CT) scans showed the presence of contusion.

**Methods:**

Fifty patients with MTBI and simultaneous brain contusion in their CT scans were enrolled according to specific exclusion criteria and studied for 14 months. The patients were followed up for two weeks after their admission for neurosurgical interventions, decreased level of consciousness and other neurological complications.

**Results:**

The presence of neurological symptoms increased the length of hospital stay and number of CT scans. Forty-two percent of MTBI patients with contusion did not have any objective neurological signs. Fifty percent returned later to the hospital with neurologic symptoms and signs. The most leading causes of the MTBI were post seizure headache and dizziness. Rehospitalization was increased in the patients with altered level of consciousness. The size of brain contusion increased in two patients without further need for neurosurgical intervention.

**Conclusions:**

Contusion alone did not worsen the prognosis of patients in short term follow up and did not cause neurosurgical interventions.

**Keywords:**

Mild traumatic brain injury, Contusion, Computed tomography, Outcome, Rehospitalization


					Volume 4, Suppl. 1 Nov 2012
Publisher: Kermanshah University of Medical Sciences
URL: http://www.jivresearch.org
Copyright:
Congress of Iranian Neurosurgeons, 14-16 November 2012, Kermanshah, Iran
Paper No. 48
Effects of brain contusion on mild traumatic brain injured
patients
Mohammad Amin Zare a
, Koorosh Ahmadi b
, Shayan Abdollah Zadegan c
, Davood Farsi a
,
Vafa Rahimi-Movaghar c,*
a
Department of Emergency Medicine, Tehran University of Medical Sciences, Tehran, Iran.
b
Department of Emergency Medicine, Alborz University of Medical Sciences, Alborz, Iran.
c
Sina Trauma and Surgery Research Center, Tehran University of Medical Sciences, Tehran, Iran.
Abstract:
Background: Traumatic brain injury (TBI) is an important health issue with high prevalence worldwide. The most
common type of TBI is mild traumatic brain injury (MTBI). MTBI is defined as a condition with self-limited symptoms;
however it could cause some structural abnormalities in brain and become more complicated. Visible structural brain
damage could have an important effect on post- MTBI recovery, but the outcome is not yet fully
understood. Therefore, the present study aims to investigate the clinical course of MTBI patients whose computed
tomography (CT) scans showed the presence of contusion.
Methods: Fifty patients with MTBI and simultaneous brain contusion in their CT scans were enrolled according to
specific exclusion criteria and studied for 14 months. The patients were followed up for two weeks after their
admission for neurosurgical interventions, decreased level of consciousness and other neurological complications.
Results: The presence of neurological symptoms increased the length of hospital stay and number of CT scans. Forty-
two percent of MTBI patients with contusion did not have any objective neurological signs. Fifty percent returned later
to the hospital with neurologic symptoms and signs. The most leading causes of the MTBI were post seizure headache
and dizziness. Rehospitalization was increased in the patients with altered level of consciousness. The size of brain
contusion increased in two patients without further need for neurosurgical intervention.
Conclusions: Contusion alone did not worsen the prognosis of patients in short term follow up and did not cause
neurosurgical interventions.
Keywords:
Mild traumatic brain injury, Contusion, Computed tomography, Outcome, Rehospitalization
* Corresponding Author at:
Vafa Rahimi-Movaghar: Associate professor of Neurosurgery, Research Deputy, Sina Trauma and Surgery Research Center, Sina Hospital, Hassan-
Abad Square, Imam Khomeini Ave, Tehran University of Medical Sciences, Tehran, Iran. Phone: (+98) 915 342 2682, (+98) 216 6757010,
Fax: (+98) 216 675 7009, Email: v_rahimi@sina.tums.ac.ir , v_rahimi@yahoo.com, (Rahimi-Movaghar V.).

				

